# Risk of antiangiogenic adverse events in metastatic colorectal cancer patients receiving aflibercept in combination with chemotherapy: A meta-analysis

**DOI:** 10.1097/MD.0000000000034793

**Published:** 2023-09-01

**Authors:** Pu Ge, Chunyan Han, Abudurousuli Reyila, Diyue Liu, Wenying Hong, Jiaxin Liu, Jinzi Zhang, Xiao Han, Xialei Li, Mengjie Huang, Siyuan Fan, Ayidana Kaierdebieke, Xiaoyu Wu, Xiaolu Huang, Weirui Guo, Siyu Liu, Ying Bian

**Affiliations:** a Institute of Chinese Medical Sciences, University of Macau, Macau, China; b State Key Laboratory of Quality Research in Chinese Medicine, University of Macau, Macau, China; c Department of Public Health and Medicinal Administration, Faculty of Health Sciences, University of Macau, Macau, China; d School of Political Science and Public Administration, Shandong University, Qingdao, China; e Xijing Hospital of Air Force Medical University, Xi’an, China; f International School of Public Health and One Health, Hainan Medical University, Haikou, China; g Faculty of Health Sciences, University of Macau, Macau, China; h Xiangya School of Nursing, Central South University, Changsha, China; i School of Humanities and Social Sciences, Harbin Medical University, Harbin, China; j The Fifth Affiliated Hospital of Sun Yat-sat University, Zhuhai, China; k School of Pharmaceutical Sciences, Shandong University, Jinan, China; l School of Public Health, Shandong University, Jinan, China; m Department of Preventive Medicine, Yanjing Medical College, Capital Medical University, Beijing, China; n School of Public Health, Xi’an Jiaotong University, Xi’an, China; o Clinically Third Series, China Medical University, Shenyang, China; p School of Clinical Medicine of Jining Medicine University, Jining, China; q Stomatology College of Shandong University, Jinan, China.

**Keywords:** adverse events, aflibercept, antiangiogenesis, meta-analysis, metastatic colorectal cancer, safety, systematic review

## Abstract

**Background:**

Aflibercept has been approved for the treatment of metastatic colorectal cancer for more than a decade, but its antiangiogenesis adverse effect profile during treatment remains unclear. This study is conducted to systematically review the risk of antiangiogenic adverse events in patients with metastatic colorectal cancer receiving aflibercept plus chemotherapy.

**Methods:**

We searched databases, including PubMed, Embase and the Cochrane Library up to September 9, 2021. Relevant randomized controlled trials (RCTs) and single-arm studies were included in the review. Statistical analyses were performed using R to calculate the summary incidence rate of antiangiogenic-related adverse events, odds ratios and 95% CIs. Heterogeneity among the included studies was assessed by subgroup analysis. Publication bias analysis and sensitivity analysis were performed to confirm the reliability of the results.

**Results:**

A total of 2889 patients from 10 studies met the inclusion criteria. The quality of the included studies was evaluated as qualified for further quantitative synthesis. In part of single-arm studies, the occurrence rates were 44.2% (95%CI, 39.7–48.7%) for hypertension, 31.3% (95% CI, 19.3–43.3%) for proteinuria, 27.3% (95%CI, 21.2–33.4%) for epistaxis, 22.5% (95%CI, 7.8–37.3%) for hemorrhage events, 8.0% (95%CI, 2.0–14 .0%) for venous thromboembolic event in all grades and 22.6% (95%CI, 19.1–26.2%) for grade III/IV hypertension, 7.4% (95%CI, 6.2–8.5%) for grade III/IV proteinuria. In part of RCT, compared to its counterpart, aflibercept containing arm was associated with the increased incidence rate in hypertension (OR:6.30, 95%CI: 3.49–11.36), proteinuria (OR:4.12, 95%CI: 1.25–13.61), epistaxis (OR:3.71, 95%CI: 2.84–4.85), III/IV hypertension (OR:7.20, 95%CI: 5.23–9.92), III/IV proteinuria (OR:5.13, 95%CI: 3.13–8.41). The funnel plot, Begg test and Egger test were carried out on the primary endpoints, III/IV hypertension rate and III/IV proteinuria rate, the result of which detected no obvious publication bias. No significant difference was observed in subgroup analysis in the primary endpoint between the subgroups stratified by treatment line (firstline or non-firstline), chemotherapy regime (FOLFIRI or others) and study design (RCTs or single-arm trials).

**Conclusion:**

The available evidence suggests that using aflibercept is associated with an increased risk of antiangiogenic adverse events compared with controls. Further studies are needed to investigate this association. In the appropriate clinical scenario, the use of aflibercept in its approved indications remains justified. However, the results of this study should be interpreted with caution, as some of the evidence comes from single-arm clinical trials.

## 1. Introduction

Colorectal cancer (CRC) is the third most common cancer in the world and the second leading cause of cancer-related death, representing a major health burden.^[1]^ More than 1 million new cases of CRC have been diagnosed worldwide each year in recent years.^[2]^ CRC tends to be asymptomatic. The majority of patients diagnosed are in the later stages of the disease, accounting for about 20% of all CRC patients.^[3]^ Targeted therapy has become an established paradigm for treating metastatic colorectal cancer (mCRC). With the development of monoclonal antibodies and soluble receptor inhibitors targeting vascular endothelial growth factor (VEGF) and epidermal growth factor receptor, survival results for individuals with mCRC have improved.^[4]^

Aflibercept, an intravenous recombinant anti-VEGF protein, was approved by the US Food and Drug Administration (FDA) in 2012 for use in combination with 5-fluorouracil, leucovorin, and irinotecan (FOLFIRI) in patients who have failed oxaliplatin-based therapy.^[5]^ However, due to its mechanism of action, aflibercept has been associated with antiangiogenic adverse events (AEs). These include hypertension, proteinuria, hemorrhage, arterial or venous thromboembolic events, cardiac AEs, reversible posterior leukoencephalopathy syndrome, and gastrointestinal (GI) perforation. Some specific types of antiangiogenic AEs, such as GI perforation, major bleeding, and thromboembolic events, may even lead to the death of its users.^[6–8]^ The antiangiogenic side effects of regimens containing aflibercept have become an important factor restricting its usage in mCRC. To date, there has been no meta-analysis that has systematically pooled and evaluated the clinical data on antiangiogenic AEs in mCRC patients treated with aflibercept in combination with chemotherapy.

This study is designed to provide guidance for the clinical use of aflibercept in the treatment of mCRC by evaluating the risk of antiangiogenic AEs associated with aflibercept in combination with chemotherapy.

## 2. Methods

The meta-analysis was in accordance with PRISMA (Preferred Reporting Items for Systematic Review and Meta-Analysis) guidelines (see Table S1, Supplemental Digital Content, http://links.lww.com/MD/J572 for details) and was also been registered with PROSPERO (International Prospective Register of Systematic Reviews, CRD42021278871).^[9]^

### 2.1. Data sources and literature searches

Two reviewers independently searched trials published before September 9, 2021 in PubMed, Embase, and the Cochrane Library. The main search terms included “metastatic colorectal cancer” and “aflibercept”; The search strategy for each database is shown in Table S2, Supplemental Digital Content, http://links.lww.com/MD/J573. References to reviews and original studies were also examined to ensure that all papers were included. Import the search results into the Endnote software (version X9) and use it to screen out duplicate articles.

Each article was reviewed independently by 2 reviewers. Inclusion criteria were as follows:

1. Participants: There are no restrictions on the patient’s gender, race, region, or nationality, but the patient is limited to over 18 years of age.
2. Interventions: The treatment group received a combination of aflibercept and chemotherapeutic drugs, with no restrictions on the dose, type, or course of chemotherapy. In the control group, other targeted drugs or placebo with chemotherapeutic drugs or only chemotherapy were utilized and the dose, type and course of chemotherapy were unlimited. Single-arm clinical studies using aflibercept in combination with chemotherapy were included in the analysis.
3. Outcomes: The number of participants with at least 1 antiangiogenesis-related AEs should be reported in the study, including hypertension, proteinuria, bleeding, arterial or venous thromboembolic event, cardiac-related AEs, reversible posterior leukoencephalopathy syndrome and GI perforation etc. Specific descriptions of the different levels of AEs are detailed in Table 1.
4. Study design: Prospective single-arm clinical trials and RCTs.
5. English-written articles with full text or the study results in clinical registration platform were available.

**Table 1:** Definition and grading of adverse events.

Term	Grade 1	Grade 2	Grade 3	Grade 4	Term definition
Hypertension	Prehypertension (systolic BP 120–139 mm Hg or diastolic BP 80–89 mm Hg)	Stage 1 hypertension (systolic BP 140–159 mm Hg or diastolic BP 90–99 mm Hg); medical intervention indicated; recurrent or persistent (>=24 hrs); symptomatic increase by > 20 mm Hg (diastolic) or to > 140/90 mm Hg if previously WNL; monotherapy indicated Pediatric: recurrent or persistent (>=24 hrs) BP > ULN; monotherapy indicated	Stage 2 hypertension (systolic BP >=160 mm Hg or diastolic BP >=100 mm Hg); medical intervention indicated; more than one drug or more intensive therapy than previously used indicated Pediatric: Same as adult	Life-threatening consequences (e.g., malignant hypertension, transient or permanent neurologic deficit, hypertensive crisis); urgent intervention indicated Pediatric: Same as adult	A disorder characterized by a pathological increase in blood pressure; a repeatedly elevation in the blood pressure exceeding 140 over 90 mm Hg.
Proteinuria	1 + proteinuria; urinary protein < 1.0 g/24 hrs	Adults: 2 + proteinuria; urinary protein 1.0–3.4 g/24 hrs; Pediatric: urine P/C (Protein/Creatinine) ratio 0.5–1.9	Adults: urinary protein >=3.5 g/24 hrs; Pediatric: urine P/C > 1.9	–	A disorder characterized by laboratory test results that indicate the presence of excessive protein in the urine. It is predominantly albumin, but also globulin.
Epistaxis	Mild symptoms; intervention not indicated	Moderate symptoms; medical intervention indicated (e.g., nasal packing, cauterization; topical vasoconstrictors)	Transfusion, radiologic, endoscopic, or operative intervention indicated (e.g., hemostasis of bleeding site)	Life-threatening consequences; urgent intervention indicated	A disorder characterized by bleeding from the nose.
Hemorrhage	Mild; intervention not indicated	Moderate symptoms; medical intervention or minor cauterization indicated	Transfusion, radiologic, endoscopic, or elective operative intervention indicated	Life-threatening consequences; urgent intervention indicated	A disorder characterized by bleeding
Thromboembolic event	Venous thrombosis (e.g., superficial thrombosis)	Venous thrombosis (e.g., uncomplicated deep vein thrombosis), medical intervention indicated	Thrombosis (e.g., uncomplicated pulmonary embolism [venous], non-embolic cardiac mural [arterial] thrombus), medical intervention indicated	Life-threatening (e.g., pulmonary embolism, cerebrovascular event, arterial insufficiency); hemodynamic or neurologic instability; urgent intervention indicated	A disorder characterized by occlusion of a vessel by a thrombus that has migrated from a distal site via the blood stream.
Reversible posterior leukoencephalopathy syndrome	Asymptomatic; clinical or diagnostic observations only; intervention not indicated	Moderate symptoms; abnormal imaging studies; limiting instrumental ADL	Severe symptoms; very abnormal imaging studies; limiting self care ADL	Life-threatening consequences; urgent intervention indicated	A disorder characterized by headaches, mental status changes, visual disturbances, and seizures associated with imaging findings of posterior leukoencephalopathy. It has been observed in association with hypertensive encephalopathy, eclampsia, and immunosuppressive and cytotoxic drug treatment. It is an acute or subacute reversible condition.
Gastrointestinal perforation	–	Symptomatic; medical intervention indicated	Severe symptoms; elective operative intervention indicated	Life-threatening consequences; urgent operative intervention indicated	A disorder characterized by a rupture in the gastrointestinal tract.

We excluded clinical trials for which raw data were not available, clinical trials for which aflibercept exposure and outcomes data were not available, animal trials, and trials containing duplicate data or abstracts.

### 2.2. Data extraction

The data extraction was carried out independently by 2 researchers, and any disagreements were resolved in a joint discussion with the third author. Extract key information from each study, such as first author, year of publication, sample size, and so on. The number of occurrences of hypertension, proteinuria, bleeding, arterial or venous thromboembolic events, cardiac-related AEs, reversible posterior leukoencephalopathy syndrome, and GI perforation was extracted for data analysis. For each type of adverse event, the number of occurrences of all grades and the number of occurrences of grade III/IV were extracted. For randomized controlled trials (RCTs), relevant data for both experimental and control groups were extracted for further analysis.

### 2.3. Quality assessment

The quality of RCTs was assessed using the Cochrane risk of bias tool.^[10]^ The quality of single-arm clinical trials was assessed by a methodological index for non-randomized studies.^[11]^

### 2.4. Statistical analysis

R version 4.0.3 was used for all statistical analyses. The data analysis was split into 2 sections. Single-arm meta-analysis pooled the incidence of each antiangiogenic adverse event in the aflibercept arm of RCTs and single-arm studies. Randomized controlled trial meta-analyses compared the risk of various antiangiogenic AEs between the experimental and control groups in RCTs, using odds ratios as indicators. In the single-arm meta-analysis, appropriate transformations, including logarithmic transformation, logit transformation, arcsin transformation, and Freeman-Turkey double arcsin transformation, were performed on the original data that did not conform to the normal distribution to stabilize the variance of the original data. The pooled rates were calculated using either a random effects model or a fixed effects model. Cochran Q test and *I*^2^ were used to evaluate the heterogeneity between studies, and *I*^2^ was used to determine whether the combined amount was suitable for the random effects model or the fixed effects model. When the heterogeneity between studies was high (*I*^2^ > 50%), the random effects model was employed; when the heterogeneity between studies was low (*I*^2^ ≤ 50%), the fixed effects model was used. This paper’ s effect size was expressed as a 95% confidence interval (CI). Otherwise, the random-effects model was used for analysis. The primary outcomes of this study were the incidence of grade III/IV hypertension and grade III/IV proteinuria in single-arm meta-analysis. Publication bias tests, sensitivity analyses, and subgroup analyses were performed only for the primary outcomes. The funnel plot examined the risk of potential publication bias and further by Begg test or Egger linear regression test. To demonstrate stability and sensitivity, sensitivity analyses were undertaken by eliminating each study one by one and then examining the influence of each study on the pooled outcomes. We also conducted subgroup analyses to better find out the source of the heterogeneity among studies and the difference of safety of aflibercept combined chemotherapy in different regimes and treatment lines. The categorical variables included different treatment lines (first line or non-first line), chemotherapy drugs (FOLFIRI or others) and study designs (single-arm clinical trial or RCT). Two-sided *P* < .05 was considered statistically significant except where otherwise specified.

## 3. Results

### 3.1. Study selection and characteristics

**Figure 1.**
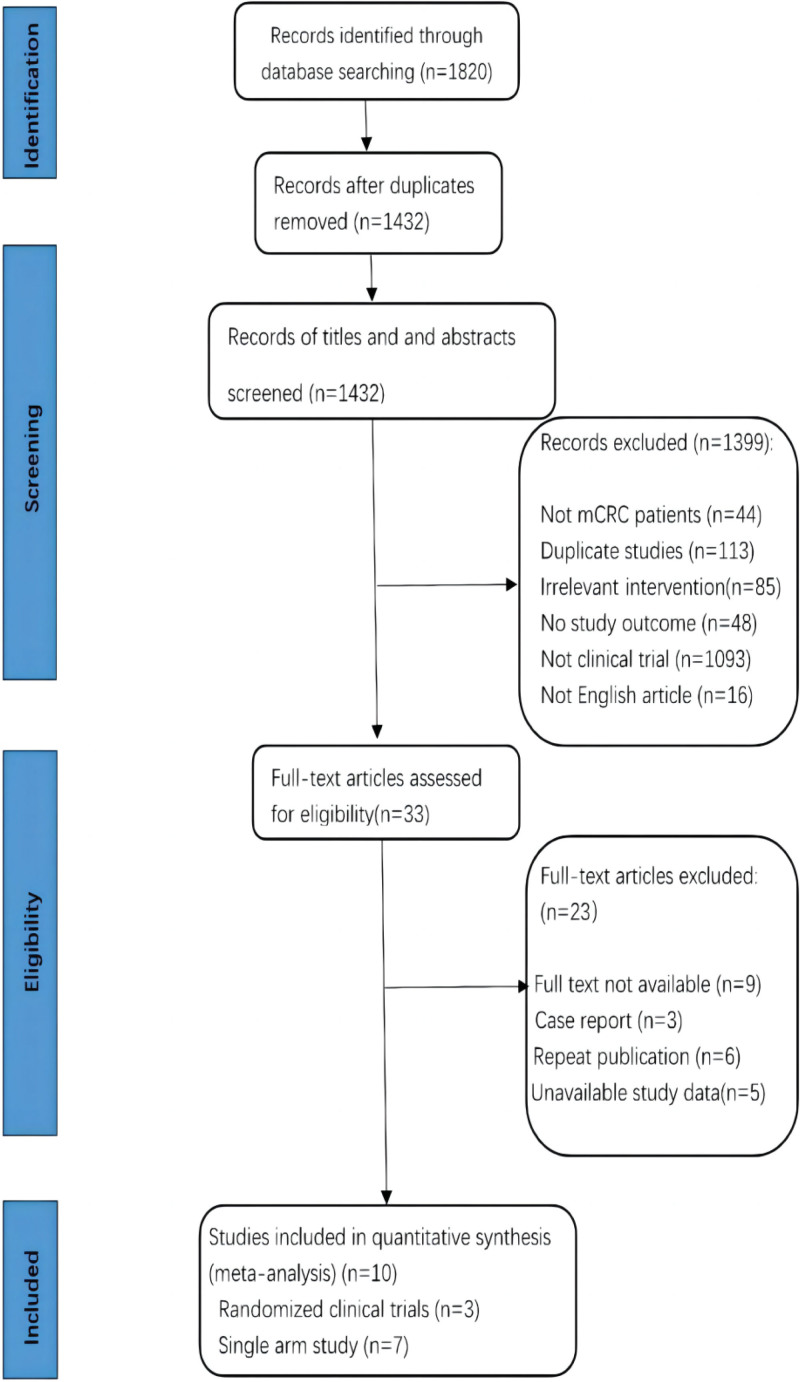
The flow diagram of study selection.

The screening process and details were shown in Figure 1, Tables S2, Supplemental Digital Content, http://links.lww.com/MD/J573 and S3, Supplemental Digital Content, http://links.lww.com/MD/J574. The literature review and identification process were shown in Figure 1. The meta-analysis evaluated the efficacy and toxicity of the aflibercept plus chemotherapy regimen for a total of 2049 patients with mCRC across 10 clinical studies.^[12–21]^ Descriptive statistical results can be seen in Table 2. (investment information of the included studies were provided in Table S4, Supplemental Digital Content, http://links.lww.com/MD/J575). The most commonly used chemotherapy was the FOLFIRI regime reported by 6 of the included studies and the other regimes include FOLFOX, modified FOLFIRI, modified FOLFOX, and capecitabine.

**Table 2:** Baseline clinical characteristics of included studies.

NO	Author	Year	Type of study	Phase	No of patients treated with Aflibercept	Male/female	Range of ECOG	Line	Median follow up (month)	Chemotherapy	Primary endpoint
1	G.Folprecht^[15]^	2016	RCT	II	119	76/43	0–2	First line	17.5	mFOLFOX6	12mPFS
2	Jin^[16]^	2018	RCT	III	223	128/95	0–1	Second line	NR	FOLFIRI	PFS
3	Eric^[17]^	2012	RCT	III	612	365/247	0–2	Second line	22.3	FOLFIRI	OS
4	John^[18]^	2019	Single-arm	II	63	37/26	NR	NR	NR	Capecitabine	2mPFS
5	Alexandra^[19]^	2020	Single-arm	II	40	17/23	0–2	First line	34.0	FOLFIRI	6mPFS
6	George^[20]^	2018	Single-arm	II	73	51/22	0–1	First line	24.5	FOLFIRI	12mPFS
7	Benoist^[21]^	2019	Single-arm	II	49	26/23	0–2	First line	22.5	mFOLFOX7	PFS
8	Alexios^[22]^	2019	Single-arm	II	31	20/11	0–1	First line	18.9	mFOLFIRI	ORR
9	Tadamichi^[23]^	2018	Single-arm	II	60	34/26	0–1	Second line	NR	FOLFIRI	ORR
10	Brigitte^[24]^	2018	Single-arm	II	779	465/314	0–2	NR	NR	FOLFIRI	NO. of AEs

12mPFS = 12-month progression-free survival rate, AES = adverse events, ECOG = eastern cooperative oncology group, NR = not reported, ORR = objective response rate, OS = overall survival, PFS **=** progression-free survival.

### 3.2. Quality assessment

In any of the 3 RCTs, there was no considerable risk of bias (see Fig. S1, Supplemental Digital Content, http://links.lww.com/MD/J576). The 7 single-arm experiments were of qualified quality for analysis (methodological index for non-randomized studies index score > 12) (see Table S5, Supplemental Digital Content, http://links.lww.com/MD/J577).

### 3.3. III/IV hypertension rate

**Figure 2.**
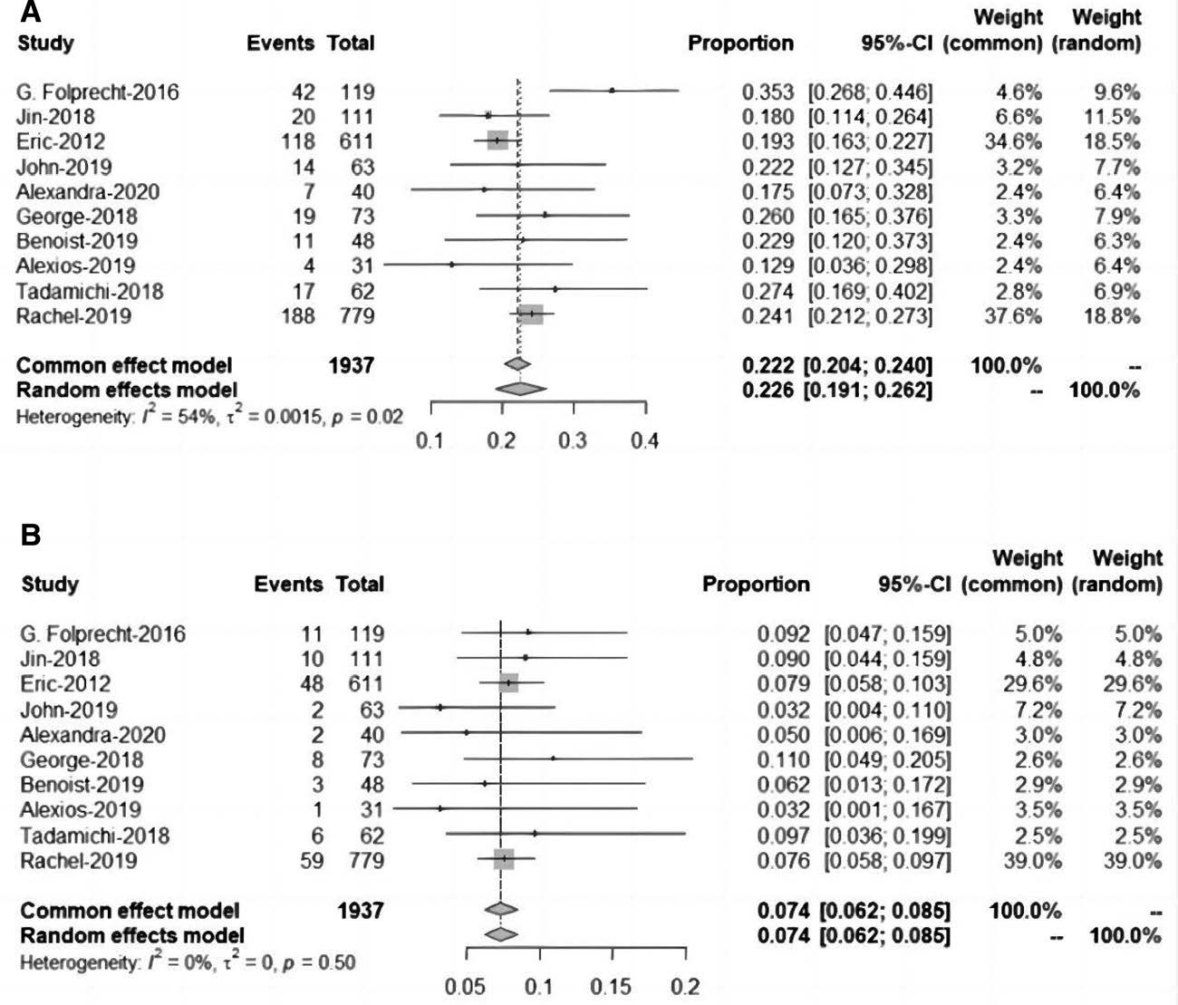
The pooled results of primary outcomes. The pooled rates of III/IV hypertension (A), and III/IV proteinuria (B) of aflibercept plus chemotherapy in the treatment of mCRC.

Original rate was used to analyze III/IV hypertension rate. All of the 10 studies reported the number of patients with III/IV hypertension involving 1937 patients in total.^[12–21]^ Because of the relatively high heterogeneity, the results were combined using a random effects model. The pooled III/IV hypertension rate was 22.6% (95%CI, 19.1–26.2%, *I*^2^ = 54%) (Fig. 2A).

### 3.4. III/IV proteinuria rate

Original rate was used to analyze III/IV proteinuria rate. All of the 10 studies reported the number of patients with III/IV proteinuria involving 1937 patients.^[12–21]^ Results were combined using a fixed effects model due to relatively low heterogeneity. the pooled III/IV proteinuria rate was 7.4% (95%CI, 6.2–8.5%, *I*^2^ = 0%) (Fig. 2B).

### 3.5. Other outcomes of single-arm meta-analysis

We combined data from the included studies on rates of each type of adverse event. Six kinds of antiangiogenic related AEs (hypertension, proteinuria, epistaxis, hemorrhage events, venous thromboembolic events, and GI perforation) and 3 kinds of III/IV antiangiogenic related AEs (III/IV hemorrhage events, III/IV venous thromboembolic events, and III/IV GI perforation) were analyzed in this part.

Among all grades of antiangiogenic AEs, the pooled risk was, in descending order, hypertension, proteinuria, epistaxis, hemorrhage events, venous thromboembolic events, and GI perforation. Using the random effects model, the rate of hypertension was 44.2%; the rate of proteinuria was 31.3%; the rate of epistaxis was 27.3%; the rate of hemorrhage events was 22.5%; and the rate of venous thromboembolic events was 8.0%. Using the fixed effects model, the rate of GI perforation was 0.6%.

Among grade III/IV AEs, the rate of III/IV hypertension was 2.6% using the fixed effects model; the rate of III/IV venous thromboembolic events was 6.5% using the random effects model; the rate of III/IV GI perforation was 0.6% using the fixed effects model.

Among all grades of AEs, hypertension had the highest incidence rate and GI perforation had the lowest incidence rate. Among grade III/IV AEs, grade III/IV hypertension had the highest incidence, and grade III/IV GI perforation had an equal incidence of all grade GI perforation and was lower than the rate of III/IV hypertension and III/IV proteinuria. The pooled outcome of AEs in part of single arm meta-analysis was available in Table 3.

**Table 3:** The pooled secondary outcomes of AEs.

Adverse event	Studies included	ES (effect size)	95%CI	*I^2^*	Model used
Hypertension	10	44.2%	39.7–48.7%	61%	Random effects model
Proteinuria	10	31.3%	19.3–43.3%	98%	Random effects model
Epistaxis	5	27.3%	21.2–33.4%	63%	Random effects model
Hemorrhage events	4	22.5%	7.8–37.3%	96%	Random effects model
Venous thromboembolic events	4	8.0%	2.0–14.0%	80%	Random effects model
Gastrointestinal perforation	4	0.6%	0.0–1.1%	25%	Fixed effects model
III/IV hemorrhage events	4	2.6%	1.4–3.9%	0%	Fixed effects model
III/IV venous thromboembolic events	4	6.5%	2.7–10.3%	68%	Random effects model
III/IV gastrointestinal perforation	4	0.6%	0.0–1.1%	25%	Fixed effects model

### 3.6. Outcomes of meta-analysis based on randomized controlled trials

**Figure 3.**
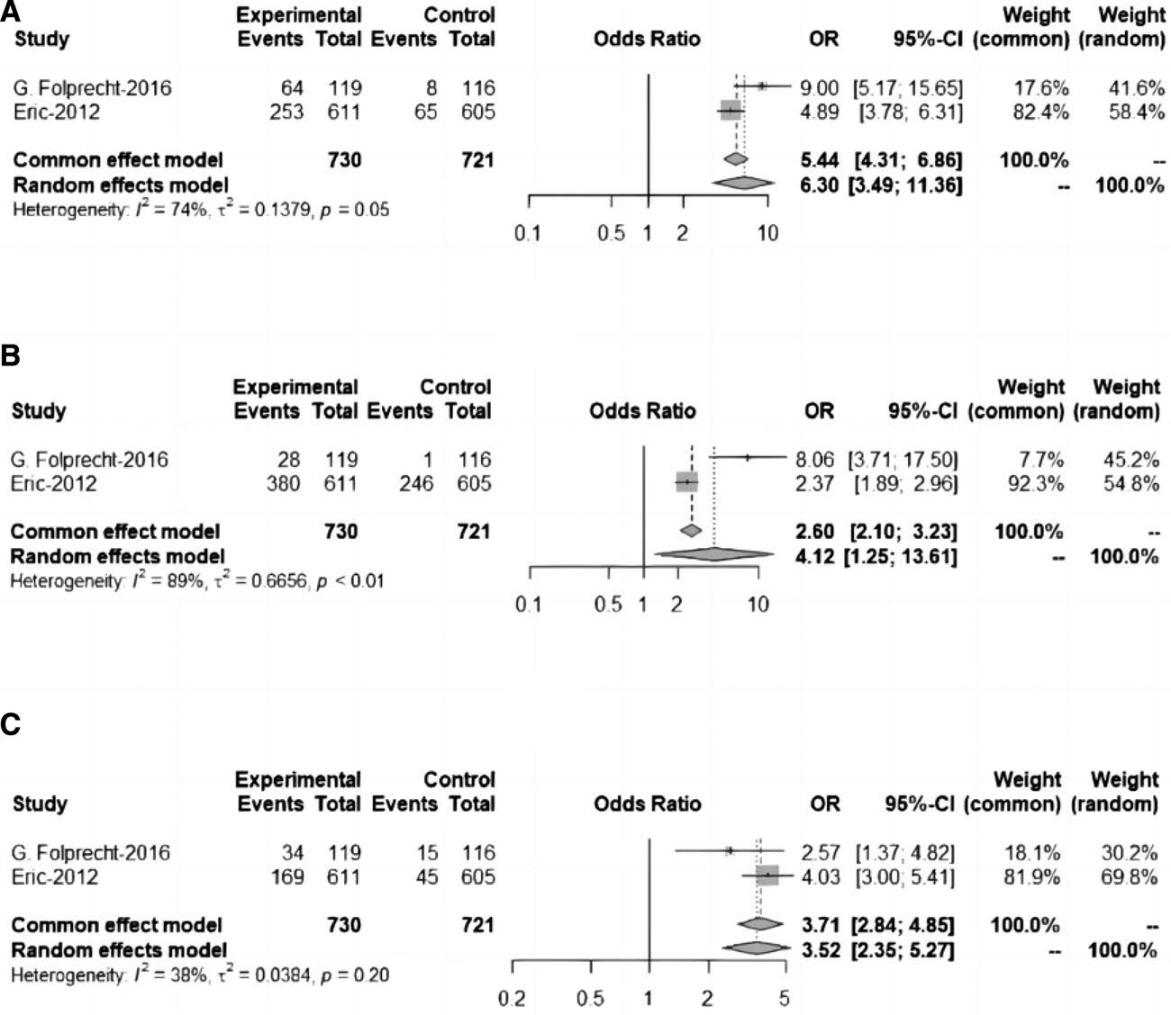
Risk of all-grade antiangiogenic-related adverse events based on randomized controlled trials. The risk of hypertension (A), proteinuria (B), and epistaxis (C).

The outcomes of meta-analysis based on RCTs are shown in Figure 3 and Table 4. Among the 3 RCTs included, 2 were analyzed in this section, while the other RCT has been analyzed in the single arm meta-analysis (in the Jin-2018 study, some subjects who should have been given a placebo were treated with aflibercept, therefore, we only used data from the aflibercept arm in this study for the single arm meta-analysis). (See Fig. 3 and Table 4 for details.)

**Table 4:** The pooled results of meta-analysis based on randomized controlled trials.

Adverse event	Studies included	Odds ratio	95%CI of odds ratio	*P*	*I^2^*	Model used
Hypertension	2	6.30	3.49–11.36	<0.001	74%	Random effects model
Proteinuria	2	4.12	1.25–13.61	0.020	89%	Random effects model
Epistaxis	2	3.71	2.84–4.85	<0.001	38%	Fixed effects model
III/IV hypertension	2	7.20	5.23–9.92	<0.001	0%	Fixed effects model
III/IV proteinuria	2	5.13	3.13–8.41	<0.001	0%	Fixed effects model

This section of the analysis included 731 patients from the 2 RCTs, with a patient age range of 27–85 years and a 60.3% male representation. Both experimental group using aflibercept and the control group using chemotherapy or placebo. Based on data of RCTs, we examined rates of hypertension, proteinuria, and epistaxis in this section.

The all-grade hypertension rate and the proteinuria rate were combined using a random effects model because of relatively high heterogeneity, and the results showed that subjects receiving aflibercept in combination with chemotherapy had a higher risk than those receiving chemotherapy (*P <* .05).

Because of the relatively low heterogeneity, the results were combined using a fixed effects model. Aflibercept in combination with chemotherapy had a higher epistaxis risk than chemotherapy (*P <* .05).

We examined rates of the grade III/IV hypertension and III/IV proteinuria in this section, and the results showed that subjects receiving aflibercept in combination with chemotherapy had a higher risk of III/IV hypertension and III/IV proteinuria than those receiving chemotherapy(*P <* .05).

### 3.7. Publication bias

**Figure 4.**
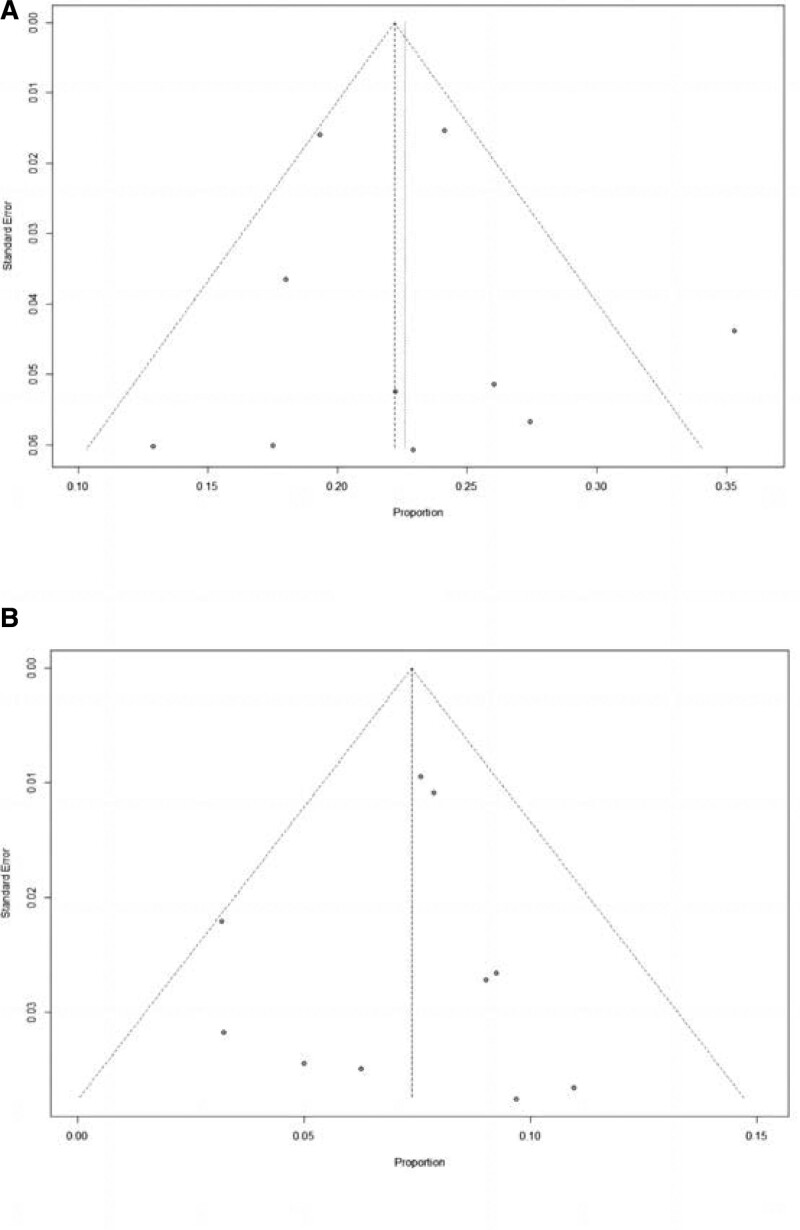
Funnel plots for primary outcomes. Funnel plots for III/IV hypertension rate (A), and III/IV proteinuria rate (B).

The funnel plots of III/IV hypertension rate and III/IV proteinuria rate were shown in Figure 4. From the funnel plot, it can be seen that there is little possibility of publication bias in this study. Begg test and Egger test were also conducted to confirm the above results (III/IV hypertension rate: Begg test, *z* = −0.45, *P* = .6547; Egger test, *t* = 0.29, df = 8, *P* = .7809; III/IV proteinuria rate: Begg test, *z* = 0.98, *P* = .3252; Egger test, *t* = −0.39, df = 8, *P* = .7042). (See Fig. 4 for details.)

### 3.8. Sensitivity analysis

**Figure 5.**
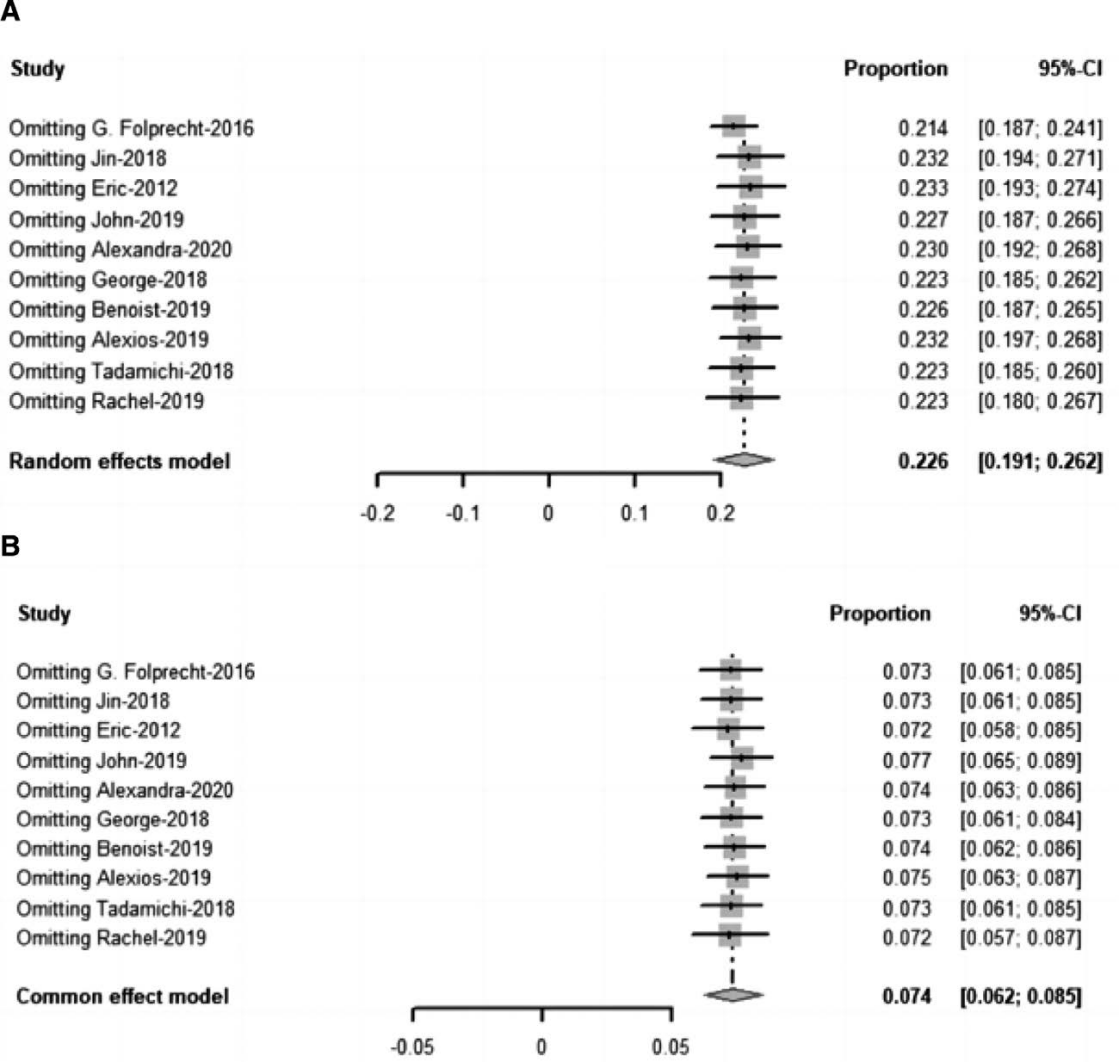
Sensitivity analyses for primary outcomes. Sensitivity analysis for III/IV hypertension rate (A), and III/IV proteinuria rate (B).

The pooled outcomes of III/IV hypertension rate and III/IV proteinuria rate were stable and no obvious bias was detected (see Fig. 5 for details).

### 3.9. Subgroup analysis

To explore the reasons for the heterogeneity of the meta-analysis, we divided subgroups according to 3 variables (treatment line, chemotherapy drug, and study design), and all subgroup effects for the III/IV hypertension rate and III/IV proteinuria rate were calculated. The results showed no significant difference in the incidence of the III/IV hypertension and III/IV proteinuria between the subgroups stratified by the above mentioned aspect (*P* > .05, see Table 5 for details).

**Table 5:** The results of subgroup analysis of main outcomes.

Subgroups		III/IV hypertension rate				III/IV proteinuria rate			
		No.	ES (95%CI)	*P*	*I* ^2^	No.	ES (95%CI)	*P*	*I* ^2^
Treatment line	Firstline	5	23.6%(15.7–31.4%)	0.660	65%	5	7.0%(4.2–9.8%)	0.792	0%
	Non-first line	5	21.7%(19.7–23.7%)		41%	5	7.4%(6.2–8.7%)		13%
Study design	RCT	3	23.7%(13.4–34.1%)	0.955	84%	3	8.2%(6.3–10.0%)		0%
	Single-arm clinical trial	7	23.4%(20.9–25.9%)		0%	7	6.8%(5.4–8.3%)	0.275	12%
Chemotherapy strategy	FOLFIRI	7	21.5%(19.6–23.5%)	0.196	41%	7	7.7%(6.4–8.9%)	0.256	0%
	Others	3	27.4%(18.5–36.3%)		57%	3	5.8%(2.8–8.8%)		36%

## 4. Discussion

Angiogenesis, the formation of new blood vessels from existing ones, is a critical process in the development and growth of human tissue. Pathological angiogenesis is a key component of cancer progression and a prerequisite for tumor metastasis. There are 2 major signaling pathways of angiogenesis, the VEGF signaling pathway and the angiopoietin signaling pathway.^[22,23]^ An important strategy to inhibit neovascularisation in solid tumors is the inhibition of the VEGF pathway, which initiates the angiogenic signaling process by increasing endothelial cell permeability and proliferation.^[23,24]^ The drugs that inhibit the VEGF signaling pathway can be divided into the following categories: The first category is monoclonal antibodies, such as bevacizumab, ramucirumab, and ranibizumab; the second category is recombinant proteins, including aflibercept, conbercept; the third category is small molecule inhibitors of the VEGF pathway, including sorafenib, vandetanib, regorafenib, and others. These small molecule inhibitors often have anti-tumor activity by inhibiting EGFR, Raf/MEK/ERK and other signaling pathways in addition to inhibiting the VEGF pathway.^[25]^ Bevacizumab, ramucirumab, aflibercept, and regorafenib are the primary anti-VEGF medications presently utilized in persons with mCRC.^[26]^

In studies, aflibercept is able to reduce cellular proliferation and in vivo growth of a variety of solid tumor cells.^[27]^ Compared to bevacizumab, aflibercept had an inherent difference regarding the inhibition of VEGF-B and placental growth factor. Increased placental growth factor expression has been shown to correlate with CRC progression and a less favorable prognosis.^[28]^ In vitro, aflibercept appeared to have greater potency and affinity than bevacizumab in blocking VEGF-A-induced activation of vascular endothelial growth factor receptor-1 and vascular endothelial growth factor receptor-2.^[29]^ Chiron M et al^[30]^ found that aflibercept had greater anti-tumor activity than bevacizumab. Aflibercept is approved for the treatment of mCRC in the United States, European Union and Japan. Clinicians typically combine aflibercept with FOLFIRI chemotherapy as second-line treatment for patients with oxaliplatin-resistant mCRC. Clinical trials of aflibercept in ovarian, prostate, pancreatic, breast, non-small cell lung, endometrial, melanoma, urothelial, glioma, thyroid, and other cancers are ongoing and may lead to the broader clinical use of this medicine in the future. (Clinical studies of aflibercept in the treatment of ovarian cancer, prostate cancer, pancreatic cancer, breast cancer, non-small cell lung cancer, endometrial cancer, melanoma, urothelial cancer, glioma, thyroid cancer, and other cancers are ongoing.)^[31–39]^ However, antiangiogenic AES have become one of the major factors limiting the clinical use of aflibercept in mCRC. We conducted the systematic review and meta-analysis to assess the risk of antiangiogenic AES in mCRC patients receiving aflibercept in combination with chemotherapy.

We included 10 clinical trials in the research.^[12–21]^ The incidence of III/IV hypertension and proteinuria were the major outcomes. The pooled incidence of III/IV hypertension was 22.6% (95%CI, 19.1–26.2%, *I*^2^ = 54%) and the pooled incidence of III/IV proteinuria was 7.4% (95%CI, 6.2–8.5%, *I*^2^ = 0%). Six kinds of antiangiogenic related AES (hypertension, proteinuria, epistaxis, hemorrhage events, venous thromboembolic events, and GI perforation) and the other 3 kinds of III/IV antiangiogenic related AES (III/IV hemorrhage events, III/IV venous thromboembolic events, and III/IV GI perforation) were also analyzed. Due to the lack of data on AES such as arterial thromboembolic events, slowed wound healing, cardiac-related AES, reversible posterior leukoencephalopathy syndrome, etc., we did not perform a meta-analysis of the incidence and risk of these antiangiogenic-related AES. In all grades of AES, the pooled incidence was as high as 44.2%. The pooled incidences of proteinuria, epistaxis, and hemorrhage events were as high as 31.3%, 27.3%, and 22.5%. The incidences of venous thromboembolic events and GI perforation were relatively low (8.0% and 0.6%). The incidence of III/IV venous thromboembolic events, III/IV hemorrhage events and III/IV GI perforation were 6.5%, 2.6%, and 0.6%. The incidence of any grade GI perforation was the same as the incidence of grade III/IV GI perforation, which means that once a GI perforation occurs, it can have serious consequences for the patient.

We also analyzed data from 2 RCTs. The risks of hypertension, proteinuria, epistaxis, III/IV hypertension, and III/IV proteinuria were analyzed. Subjects who received aflibercept in combination with chemotherapy had a higher risk of the abovementioned AES than those who received chemotherapy, suggesting that the antiangiogenesis-related AES of aflibercept need to be monitored by clinicians and others. The addition of aflibercept to chemotherapy was expected to increase the likelihood of antiangiogenic AES in patients, and our findings back this up. Therefore, patients receiving chemotherapy in combination with aflibercept should be aware that while this therapy improves the survival benefit for patients, it also increases the risk of AES for patients.

The development of hypertension can be attributed to the inhibition of VEGF, which raises blood pressure via 2 main pathways. One is that VEGF mediates the production of nitric oxide, which is a classic type of vasodilator, causing vasoconstriction due to an imbalance between vasodilator and vasoconstrictor; another is that VEGF dilates arterioles and venules to lower blood pressure, therefore inhibiting VEGF induces increased peripheral resistance and hypertension.^[40,41]^ In cases of uncontrolled hypertension, blood pressure should be closely monitored, and antihypertensive medication should be used.^[42]^

The underlying pathogenesis of proteinuria has been linked to the function of VEGF in kidney tissue; inhibition of VEGF results in decreased numbers of epithelial cells and increased permeability, allowing protein flux from the urine as a result.^[43,44]^ Relevant study points out that the occurrence time of proteinuria was the second or third week of the treatment process, and the kidney function may recover to normal if the administration of the drug were stopped on time on the onset of kidney damage.^[45]^ For early detection of proteinuria, urine protein and creatinine spot tests are recommended. Patients with primary renal lesions should be carefully monitored before drug administration and during treatment. According to FDA guidelines, if the protein in urine exceeds 1 g/24 h, the anti-VEGF agents should be suspended until the urine protein level returns to normal.^[46]^ If urine protein exceeds 2 g/24 h, the medicine should be stopped, according to the instructions for usage.

Studies have shown that the pathogenesis of bleeding and arterial or venous thromboembolic events is complex and still unknown. Some hypotheses have been proposed regarding the function of VEGF, which is an essential factor in the proliferation and survival of new epithelial cells in newly formed vessels. Inhibition of VEGF induces a reduction in the number of epithelial cells, resulting in the underlying matrix of epithelial cells being exposed, hemorrhagic and thrombotic events will occur as a result.^[47,48]^ With regard to bleeding events, the US FDA has recommended that the signs and symptoms of severe GI and other bleeding should be closely monitored. Patients with a high-grade bleeding event should not be treated with aflibercept.^[49]^ For patients with a history or high risk of venous thromboembolism, prophylactic low-molecular-weight heparin has been recommended, with careful assessment of the balance between thrombosis and bleeding.^[50]^

Despite a relatively low incidence rate, GI perforation is one of the fatal AEs that requires greater attention from clinicians in practice. Although the pathogenesis remains unclear and there is no specific guideline for the management of anti-VEGF-induced GI perforation, the following options may help to reduce the mortality of patients with GI perforation.^[7]^ Identification of individuals at high risk of GI perforation with a history of GI ulcer, diverticulosis, endoscopic evaluation, GI obstruction, and previous surgery is a crucial initial step in minimizing the incidence of GI perforation. If GI is detected, prompt surgery, fluid resection, and broad-spectrum antibiotics are warranted simultaneously.^[51]^

Some meta-analyses were conducted to explore the occurrence rate of specific type of AEs in tumor patients treated with aflibercept. The occurrence rate of all-grade hypertension, all-grade proteinuria, all-grade GI perforation and all-grade hemorrhagic events were 42.4% (95%CI: 35.0–50.3), 33.9% (95%CI: 27.3–42.1), 1.9% (95%CI: 1.0–3.8 %) and 22.1% (95%CI: 16.5–29.7), respectively.^[7,8]^ The incidence rate of high-grade hypertension, high-grade proteinuria and high-grade hemorrhagic were 17.4 % (95%CI: 13.7–21.9), 7.9% (95%CI: 6.1–10.2), and 4.2%(95%CI: 3.9–4.6), respectively.^[8,49,52,53]^ Compared with placebo or treatment without aflibercept, aflibercept increased the incidence of hypertension, proteinuria, hemorrhagic events, GI perforation and hemorrhagic events in all-grade, and high-grade bleeding.^[7,8,49,52]^ Aflibercept increased high-grade infections compared to the control group as the author did not pool the outcomes of all-grade infections. Bevacizumab, another molecularly targeted the anti-VEGF pathway agent, was compared with aflibercept in some of the above meta-analyses. Compared with bevacizumab, aflibercept increased the incidence of hypertension and proteinuria, while there was no statistical significance between these 2 agents in the incidence of hemorrhagic events.^[54]^

Based on the current study, we propose the following recommendation in clinical practice, aflibercept in combination with chemotherapy should only be used in mCRC patients who are suitable for this therapy, after appropriate evaluation, under the supervision of a physician with sufficient knowledge and experience, in a medical setting that can adequately respond to an emergency. In the treatment of mCRC, clinicians should fully explain the benefits and risks to patients or their families, based on the existing clinical evidence, and select this therapy for clinical treatment after obtaining the consent of patients or their families, based on their conditions and wishes. Patients receiving the therapy should be monitored with regular blood pressure measurements, urine protein and blood images. Serious antiangiogenic related AES including thromboembolic events, GI perforation, severe GI bleeding, severe hypertension, and severe proteinuria once appear, administration of aflibercept should be suspended and related condition should be managed in a timely manner. In cases where the patient has experienced GI perforation or severe GI bleeding, the therapy should be discontinued and reconsidered for future use. If patients require surgery, such as resection of liver metastases, aflibercept should be discontinued before the surgery.

The current meta-analysis has several limitations:

1. The meta-analysis included only literature published in English, which may lead to language bias.
2. The number of studies included in the meta-analysis was limited. There were few head-to-head RCTs and some methodological flaws. We conducted both the single-arm meta-analysis and the RCT-based meta-analysis. Compared with the RCT-based meta-analysis, the single-arm meta-analysis had lower statistical power and accuracy. However, our RCT-based meta-analysis comprised just 2 RCTs. The credibility of the results is affected by the small number of included trials.
3. Because the included trials did not provide data on tumor location, race, and RAS/BRAF expression, we were not able to analyze the relationship between these factors and the efficacy and safety of aflibercept.
4. We found remarkable heterogeneity, clinical heterogeneity and methodological heterogeneity in the current study. Clinical heterogeneity included differences in patients’ clinical data (disease severity, race, cancer cell genotype, and so on), treatment line (first-line and second-line), different chemotherapy regimens, different outcome indicators, and different study designs.
5. All included trials were funded by the pharmaceutical industry, which could lead to potential bias due to financial conflict of interest.
6. This is a meta-analysis based on trial results, rather than individual data, which may prevent the analysis from adjusting for confounding factors and further investigating the specific population that may experience fewer AEs with aflibercept-containing therapy.

## 5. Conclusion

In conclusion, this study shows that the use of aflibercept is associated with a significantly increased risk of antiangiogenic AES. Despite the current findings, it is crucial to remember that the usage of aflibercept greatly improves the outcome of CRC patients. Its use in these patients should be continued under the appropriate clinical scenario. Further studies are recommended to investigate the risk factors for antiangiogenic AES and to prevent antiangiogenic AES linked with aflibercept medication, such as bleeding and GI perforation. Clinicians need to be aware of the risks associated with the use of aflibercept and ensure rigorous monitoring to further improve patient outcomes.

## Author contributions

**Conceptualization:** Pu Ge.

**Data curation:** Pu Ge, Abudurousuli Reyila.

**Formal analysis:** Pu Ge, Chunyan Han, Abudurousuli Reyila.

**Methodology:** Pu Ge.

**Software:** Pu Ge.

**Writing – original draft:** Pu Ge, Chunyan Han, Abudurousuli Reyila, Diyue Liu, Wenying Hong, Jiaxin Liu, Jinzi Zhang, Xiao Han, Xialei Li, Mengjie Huang, Siyuan Fan, Ayidana Kaierdebieke, Xiaoyu Wu, Xiaolu Huang, Weirui Guo, Siyu Liu, Ying Bian.

**Writing – review & editing:** Pu Ge, Wenying Hong, Jiaxin Liu, Jinzi Zhang, Xiao Han, Xialei Li, Mengjie Huang, Siyuan Fan, Ayidana Kaierdebieke, Xiaoyu Wu, Xiaolu Huang, Weirui Guo, Siyu Liu, Ying Bian.

## Supplementary Material
