# Supplementary material for: Risk of antiangiogenic adverse events in metastatic colorectal cancer patients receiving aflibercept in combination with chemotherapy: A meta-analysis
Source: Medicine (Baltimore). 2023 Sep 1;102(35):e34793. doi: 10.1097/MD.0000000000034793 (PMC10476758; doi:10.1097/MD.0000000000034793)
Supplement: Supplementary file 2 [file medi-102-e34793-s002.pdf]

Supplementary Table 2 Search Strategy

Search date	9 September 2021
Pubmed	#1 (aflibercept[mh]) OR aflibercept OR (VEGF Trap-regeneron) OR VEGF-Trap OR (VEGF Trap) OR (VEGF Trap-Eye) OR eylea OR Zaltrap OR (AVE 0005) OR AVE-0005 OR (AVE 005) OR AVE-005 OR AVE0005 OR AVE005 OR ZIV-aflibercept<br><br>#2 (Colorectal Neoplasms[mh]) OR (Colorectal Neoplasm) OR (Neoplasm, Colorectal) OR (Neoplasms, Colorectal) OR (Colorectal Tumors) OR (Colorectal Tumor) OR (Tumor, Colorectal) OR (Tumors, Colorectal) OR (Colorectal Cancer) OR (Cancer, Colorectal) OR (Cancers, Colorectal) OR (Colorectal Cancers) OR (Colorectal Carcinoma) OR (Carcinoma, Colorectal) OR (Carcinomas, Colorectal) OR (Colorectal Carcinomas) OR (Colonic Neoplasms[mh]) OR (Colonic Neoplasm) OR (Neoplasm,Colonic) OR (Neoplasms, Colonic) OR (Colon Neoplasms) OR (Colon Neoplasm) OR (Neoplasm,Colon) OR (Neoplasms,Colon) OR (Cancer of Colon) OR (Colon Cancers) OR (Cancer of the Colon) OR (Colonic Cancer) OR (Cancer, Colonic) OR (Cancers,Colonic) OR (Colonic Cancers) OR (Colon Cancer) OR (Cancer, Colon) OR (Cancers,Colon) OR (Rectal Neoplasms[mh]) OR (Neoplasm,Rectal) OR (Rectal Neoplasm) OR (Rectum Neoplasms) OR (Neoplasm,Rectum) OR (Rectum Neoplasm) OR (Rectal Tumors) OR (Rectal Tumor) OR (Tumor,Rectal) OR (Neoplasms,Rectal) OR (Cancer of Rectum) OR (Rectum Cancers) OR (Rectal Cancer) OR (Cancer,Rectal) OR (Rectal Cancers) OR (Rectum Cancer) OR (Cancer,Rectum) OR (Cancer of the Rectum)<br><br>#3 animals [mh] NOT humans [mh]<br><br>#4 #1 AND #2<br>#5 #4 NOT #3
Cochrane	#1 MeSH descriptor: [Colorectal Neoplasms] explode all trees

	#2 MeSH descriptor: [Rectal Neoplasms] explode all trees<br>#3 MeSH descriptor: [Colonic Neoplasms] explode all trees<br>#4 (colon):ti,ab,kw (Word variations have been searched)<br>#5 (Taenia Coli):ti,ab,kw (Word variations have been searched)<br>#6 (Appendix Epiploica):ti,ab,kw (Word variations have been searched)<br>#7 (Omental Appendix):ti,ab,kw (Word variations have been searched)<br>#8 (Appendix, Omental):ti,ab,kw (Word variations have been searched)<br>#9 (Omental Appendices):ti,ab,kw (Word variations have been searched)<br>#10 (Appendices, Omental):ti,ab,kw (Word variations have been searched)<br>#11 (Rectum):ti,ab,kw (Word variations have been searched)<br>#12 (Rectums):ti,ab,kw (Word variations have been searched)<br>#13 (Colorectal*):ti,ab,kw (Word variations have been searched)<br>#14 (Colonic*):ti,ab,kw (Word variations have been searched)<br>#15 (Rectal*):ti,ab,kw (Word variations have been searched)<br>#16 (Large Intestine*):ti,ab,kw (Word variations have been searched)<br>#17 (Intestine, Large*):ti,ab,kw (Word variations have been searched)<br>#18 (Neoplasms):ti,ab,kw (Word variations have been searched)<br>#19 (Neoplas*):ti,ab,kw (Word variations have been searched)<br>#20 (Carcinoma*):ti,ab,kw (Word variations have been searched)<br>#21 (Neoplasia):ti,ab,kw (Word variations have been searched)<br>#22 (Neoplasias):ti,ab,kw (Word variations have been searched)<br>#23 (Neoplasm):ti,ab,kw (Word variations have been searched)

	#24 (Tumors):ti,ab,kw (Word variations have been searched)<br>#25 (Tumor):ti,ab,kw (Word variations have been searched)<br>#26 (Cancer):ti,ab,kw (Word variations have been searched)<br>#27 (Cancers):ti,ab,kw (Word variations have been searched)<br>#28 (Malignancy):ti,ab,kw (Word variations have been searched)<br>#29 ("malignancies"):ti,ab,kw (Word variations have been searched)<br>#30 (Malignant Neoplasms):ti,ab,kw (Word variations have been searched)<br>#31 (Malignant Neoplasm):ti,ab,kw (Word variations have been searched)<br>#32 (Neoplasm, Malignant):ti,ab,kw (Word variations have been searched)<br>#33 (Neoplasms, Malignant):ti,ab,kw (Word variations have been searched)<br>#34 (Benign Neoplasms):ti,ab,kw (Word variations have been searched)<br>#35 (Neoplasms, Benign):ti,ab,kw (Word variations have been searched)<br>#36 (Benign Neoplasm):ti,ab,kw (Word variations have been searched)<br>#37 (Neoplasm, Benign):ti,ab,kw (Word variations have been searched)<br>#38 #4 OR #5 OR #6 OR #7 OR #8 OR #9 OR #10 OR #11 OR #12 OR #13 OR #14 OR #15 OR #16 OR #17<br>#39 #18 OR #19 OR #20 OR #21 OR #22 OR #23 OR #24 OR #25 OR #26 OR #27 OR #28 OR #29 OR #30 OR #31 OR #32 OR #33 OR #34 OR #35 OR #36 OR #37<br>#40 #38 AND #39<br>#41 #40 OR #1 OR #2 OR #3<br>#42 (aflibercept):ti,ab,kw (Word variations have been searched)<br>#43 (VEGF Trap-regeneron):ti,ab,kw (Word variations have been searched)<br>#44 (VEGF-Trap):ti,ab,kw (Word variations have been searched)<br>#45 (VEGF Trap):ti,ab,kw (Word variations have

	been searched)<br>#46 (VEGF Trap-Eye):ti,ab,kw (Word variations have been searched)<br>#47 (eylea):ti,ab,kw (Word variations have been searched)<br>#48 (Zaltrap):ti,ab,kw (Word variations have been searched)<br>#49 (AVE 0005):ti,ab,kw (Word variations have been searched)<br>#50 (AVE-0005):ti,ab,kw (Word variations have been searched)<br>#51 (AVE 005):ti,ab,kw (Word variations have been searched)<br>#52 (AVE-005):ti,ab,kw (Word variations have been searched)<br>#53 (AVE0005):ti,ab,kw (Word variations have been searched)<br>#54 (AVE005):ti,ab,kw (Word variations have been searched)<br>#55 (ZIV-aflibercept):ti,ab,kw (Word variations have been searched)<br>#56 #42 OR #43 OR #44 OR #45 OR #46 OR #47 OR #48 OR #49 OR #50 OR #51 OR #52 OR #53 OR #54 OR #55<br>#57 #41 AND #56
Embase	('aflibercept'/exp OR 'aflibercept':ab,ti OR 'VEGF Trap-regeneron':ab,ti OR 'VEGF-Trap':ab,ti OR 'VEGF Trap' OR 'VEGF Trap-Eye':ab,ti OR 'eylea':ab,ti OR 'Zaltrap':ab,ti OR 'AVE 0005':ab,ti OR 'AVE-0005':ab,ti OR 'AVE 005':ab,ti OR 'AVE-005':ab,ti OR 'AVE0005':ab,ti OR 'AVE005':ab,ti OR 'ZIV-aflibercept':ab,ti) AND (('colorectal tumor'/exp OR 'colorectal neoplasms':ti,ab OR 'colorectal neoplasm':ti,ab OR 'neoplasm, colorectal':ti,ab OR 'neoplasms, colorectal':ti,ab OR 'colorectal tumors':ti,ab OR 'tumor, colorectal':ti,ab OR 'tumors, colorectal':ti,ab OR 'colorectal cancer':ti,ab OR 'cancer, colorectal':ti,ab OR 'cancers, colorectal':ti,ab OR 'colorectal cancers':ti,ab OR 'colorectal carcinoma':ti,ab OR 'carcinoma, colorectal':ti,ab OR 'carcinomas, colorectal':ti,ab OR 'colorectal carcinomas':ti,ab) OR ('colon tumor'/exp OR 'colonic neoplasms':ab,ti OR

	'colonic neoplasm':ab,ti OR 'neoplasm,colonic':ab,ti OR 'neoplasms, colonic':ab,ti OR 'colon neoplasms':ab,ti OR 'colon neoplasm':ab,ti OR 'neoplasm,colon':ab,ti OR 'neoplasms,colon':ab,ti OR 'cancer of colon':ab,ti OR 'colon cancers':ab,ti OR 'cancer of the colon':ab,ti OR 'colonic cancer':ab,ti OR 'cancer, colonic':ab,ti OR 'cancers,colonic':ab,ti OR 'colonic cancers':ab,ti OR 'colon cancer':ab,ti OR 'cancer, colon':ab,ti OR 'cancers,colon':ab,ti) OR ('rectum tumor'/exp OR 'rectal neoplasms':ab,ti OR 'neoplasm,rectal':ab,ti OR 'rectal neoplasm':ab,ti OR 'rectum neoplasms':ab,ti OR 'neoplasm,rectum':ab,ti OR 'rectum neoplasm':ab,ti OR 'rectal tumors':ab,ti OR 'rectal tumor':ab,ti OR 'tumor,rectal':ab,ti OR 'neoplasms,rectal':ab,ti OR 'cancer of rectum':ab,ti OR 'rectum cancers':ab,ti OR 'rectal cancer':ab,ti OR 'cancer,rectal':ab,ti OR 'rectal cancers':ab,ti OR 'rectum cancer':ab,ti OR 'cancer,rectum':ab,ti OR 'cancer of the rectum':ab,ti) OR ('colon tumor'/exp OR 'colonic neoplasms':ab,ti OR 'colonic neoplasm':ab,ti OR 'neoplasm,colonic':ab,ti OR 'neoplasms, colonic':ab,ti OR 'colon neoplasms':ab,ti OR 'colon neoplasm':ab,ti OR 'neoplasm,colon':ab,ti OR 'neoplasms,colon':ab,ti OR 'cancer of colon':ab,ti OR 'colon cancers':ab,ti OR 'cancer of the colon':ab,ti OR 'colonic cancer':ab,ti OR 'cancer, colonic':ab,ti OR 'cancers,colonic':ab,ti OR 'colonic cancers':ab,ti OR 'colon cancer':ab,ti OR 'cancer, colon':ab,ti OR 'cancers,colon':ab,ti) OR 'carcinoma of large intestine':ab,ti))
