# Supplementary material for: Risk of antiangiogenic adverse events in metastatic colorectal cancer patients receiving aflibercept in combination with chemotherapy: A meta-analysis
Source: Medicine (Baltimore). 2023 Sep 1;102(35):e34793. doi: 10.1097/MD.0000000000034793 (PMC10476758; doi:10.1097/MD.0000000000034793)
Supplement: Supplementary file 3 [file medi-102-e34793-s003.pdf]

Supplementary Table 3 Studies excluded from rescreening

Serial number	Citation format	Reasons for exclusion
1	Nct. Aflibercept and 5-FU vs. FOLFOX as 1st Line Treatment for Elderly or Frail Elderly Patients With Met. Colorectal Cancer[J]. https://clinicaltrials.gov/show/NCT03530267,2018.	Full text not avaliable
2	R Lakomý.　(2014). Aflibercept. metastatic colorectal cancer: at least as poorly tolerated as bevacizumab. Prescrire International, 23 (152), 205.	Full text not avaliable
3	Euctr, S. E.　(2007). "A Multinational, Randomized, Double-blind Study, Comparing the Efficacy of Aflibercept Once Every 2 Weeks versus Placebo in Patients with Metastatic Colorectal Cancer (MCRC) Treated with Irinotecan / 5-FU Combination　(FOLFIRI) after failure of an oxaliplatin based regimen. - VELOUR." http://www.who.int/trialsearch/Trial2.aspx?TrialID=EUCTR2007-000820-42-SE.	Full text not avaliable
4	Euctr FR. Study to evaluate aflibercept　(efficacity and toxicity) with a chemotherapy by 5 FU in first line of treatment for patients with a metastatic colorectal cancer[J]. https://trialsearch.who.int/Trial2.aspx?TrialID=EUCTR2014-001837-10-FR, 2015.	Full text not avaliable
5	Euctr GB. A multicentre randomised phase II study of aflibercept plus chemotherapy in patients with colorectal liver-only metastases deemed to be inoperable or unsuitable for upfront liver resection[J]. https://trialsearch.who.int/Trial2.aspx?TrialID=EUCTR2014-000845-64-GB, 2015.	Full text not avaliable

6	Euctr DE. Comparison of a standard chemotherapy (FOLFOX) with a combination of Aflibercept and 5FU for elderly or frail elderly patients with metastatic colorectal cancer[J]. https://trialsearch.who.int/Trial2.aspx?TrialID=EUCTR2017-000329-11-DE, 2017.	Full text not avaliable
7	Nct (2017). "mFOLFOX6 vs. mFOLFOX6 + Aflibercept as Neoadjuvant Treatment in MRI-defined T3-rectal Cancer." https://clinicaltrials.gov/show/NCT03043729.	Full text not avaliable
8	Euctr DE. Combination chemotherapy mFOLFOX6 versus combination chemotherapy and Aflibercept in patients with locally advanced rectal cancer staged T3 in MRI[J]. https://trialsearch.who.int/Trial2.aspx?TrialID=EUCTR2015-002773-38-DE, 2016.	Full text not avaliable
9	Legoux, J. L. , Malicot, K. L. , Faroux, R. , Boige, V. , Aparicio, T. (2016). Prodige 25 (ffcd 11-01) - phase ii randomized trial evaluating aflibercept associated with lv5fu2 regimen as first line treatment of non-resectable metastatic colorectal cancers (folfa). Annals of Oncology, 27 (suppl_6).	Full text not avaliable
10	Liberko, M. and R. Soumarová (2019). "Complete remission during FOLFIRI + aflibercept (Zaltrap) treatment in patient with liver and lung metastasis from colorectal cancer." Onkologie (Czech Republic) 13 (5): 237-242.	Case report
11	Ghiringhelli F, Vincent J, Beltjens F, et al. Fluorouracil, leucovorin and irinotecan associated with aflibercept can induce microscopic colitis in metastatic colorectal cancer patients[J]. Investigational new drugs, 2015, 33 (6): 1263-1266.	Case report
12	De Divitiis C, Cassata A, Nasti G, Ottaiano A, Nappi A, Barretta ML, Iaffaioli RV. Tardiva risposta completa dopo lunga remissione da trattamento con schedula FOLFIRI-aflibercept per cancro	Case report

	colorettale metastatico dopo progressione a schedula folfox-bevacizumab [Late complete response after long remission from treatment with FOLFIRI-aflibercept for metastatic colorectal cancer after progression to FOLFOX-bevacizumab]. Recenti Prog Med. 2015 Aug;106 (8):e407-13. Italian. doi: 10.1701/1960.21312. PMID: 26228865.	
13	Mitchell E P, Guarino M J, Andria M L, et al. North American (NA) subgroup results from VELOUR: Ziv-aflibercept versus placebo plus FOLFIRI in mCRC that is resistant to or has progressed after an oxaliplatin-containing regimen[J]. 2013.	Repeat publication
14	Tabernero J, Van Cutsem E, Lakomý R, et al. Aflibercept versus placebo in combination with fluorouracil, leucovorin and irinotecan in the treatment of previously treated metastatic colorectal cancer: prespecified subgroup analyses from the VELOUR trial[J]. European journal of cancer, 2014, 50 (2): 320-331.	Repeat publication
15	Ruff P, Ferry DR, Lakomỳ R, Prausová J, Van Hazel GA, Hoff PM, Cunningham D, Arnold D, Schmoll HJ, Moiseyenko VM, McKendrick JJ, Ten Tije AJ, Vishwanath RL, Bhargava P, Chevalier S, Macarulla T, Van Cutsem E. Time course of safety and efficacy of aflibercept in combination with FOLFIRI in patients with metastatic colorectal cancer who progressed on previous oxaliplatin-based therapy. Eur J Cancer. 2015 Jan;51 (1):18-26. doi: 10.1016/j.ejca.2014.10.019. Epub 2014 Nov 14. PMID: 25466509.	Repeat publication
16	Ruff P, Van Cutsem E, Lakomy R, Prausova J, van Hazel GA, Moiseyenko VM, Soussan-Lazard K, Dochy E, Magherini E, Macarulla T, Papamichael D. Observed benefit and safety of aflibercept in elderly patients with metastatic colorectal cancer: An	Repeat publication

	age-based analysis from the randomized placebo-controlled phase III VELOUR trial. J Geriatr Oncol. 2018 Jan;9 (1):32-39. doi: 10.1016/j.jgo.2017.07.010. Epub 2017 Aug 12. PMID: 28807738.	
17	Li J, Xu R, Qin S, Liu T, Pan H, Xu J, Bi F, Lim R, Zhang S, Ba Y, Bai Y, Fan N, Tsuji A, Yeh KH, Ma B, Wei V, Shi D, Magherini E, Shen L. Aflibercept plus FOLFIRI in Asian patients with pretreated metastatic colorectal cancer: a randomized Phase III study. Future Oncol. 2018 Aug;14 (20):2031-2044. doi: 10.2217/fon-2017-0669. Epub 2018 Aug 17. Erratum in: Future Oncol. 2019 Feb;15 (4):451. PMID: 30117334.	Repeat publication
18	Ruff P, Ferry D, Papamichael D, et al. Observed benefit of aflibercept in mCRC patients≥ 65 years old: results of a prespecified age-based analysis of the VELOUR study[J]. Annals of Oncology, 2013, 24: iv18.	Repeat publication
19	Nct. PERMAD: Personalized Marker-driven Early Switch to Aflibercept in Patients With Metastatic Colorectal Cancer[J]. https://clinicaltrials.gov/show/NCT02331927, 2014.	Unavailable study data
20	Euctr, A. T. (2015). "Personalized marker-driven early switch to aflibercept in patients with metastatic colorectal cancer. A run in marker determination phase followed by a marker-driven randomized part - a multicenter, multinational, two-part, phase II trial." http://www.who.int/trialsearch/Trial2.aspx?TrialID=EUCTR2012-005657-24-AT.	Unavailable study data
21	Euctr GR. Phase II Trial in Metastatic Colorectal Cancer With FOLFIRI Plus Aflibercept as First Line Treatment (MINOAS)[J]. https://trialsearch.who.int/Trial2.aspx?TrialID=EUCTR2014-001422-16-GR, 2015.	Unavailable study data
22	Legoux J L, Le Malicot K, Faroux R, et al. PRODIGE 25 (FFCD	Unavailable study

	11-01)-Phase II randomized trial evaluating aflibercept associated with LV5FU2 regimen as first line treatment of non-resectable metastatic colorectal cancers (FOLFA)[J]. Annals of Oncology, 2016, 27: vi204.	data
23	Euctr ES. Study to assess the efficacy and safety of treatment with FOLFIRI-aflibercept compared to initial treatment with FOLFIRI-aflibercept (for 6 cycles) followed by maintenance with 5FU-aflibercept, in an elderly population with metastatic colorectal cancer after failure of an oxaliplatin-based regimen[J]. https://trialsearch.who.int/Trial2.aspx?TrialID=EUCTR2016-004076-21-ES, 2017.	Unavailable study data
