# Supplementary material for: Risk of antiangiogenic adverse events in metastatic colorectal cancer patients receiving aflibercept in combination with chemotherapy: A meta-analysis
Source: Medicine (Baltimore). 2023 Sep 1;102(35):e34793. doi: 10.1097/MD.0000000000034793 (PMC10476758; doi:10.1097/MD.0000000000034793)
Supplement: Supplementary file 4 [file medi-102-e34793-s004.pdf]

Supplementary Table 4 Investment Information

Included studies	Financing body	Sponsor protocol code
16 Folprecht-2016	Sanofi (Cambridge, MA)	
	Regeneron Pharmaceuticals (Tarrytown, NY)	
17 Jin-2018	Sanofi	
18 Eric-2012	Sanofi	
	Amgen	
19 John-2019	Sanofi Genzyme	
20 Alexandra-2020	Fédération Francophone de Cancérologie Digestive (FFCD)	
	Sanofi	
21 George-2018	Sanofi	AFLIBL06723
22 Benoist-2019	Sanofi	
23 Alexios-2019	Hellenic Oncology Research Group (HORG)	
	Sanofi (Paris, France)	
	Biomedical Research (CABR)	

24<br>Tadamichi-2019	Sanofi	
25<br>EU Clinical Trials Register	Sanofi aventis recherche & développement	AFLIBC06097
