# Supplementary figures and images for: Risk of antiangiogenic adverse events in metastatic colorectal cancer patients receiving aflibercept in combination with chemotherapy: A meta-analysis
Source: Medicine (Baltimore). 2023 Sep 1;102(35):e34793. doi: 10.1097/MD.0000000000034793 (PMC10476758; doi:10.1097/MD.0000000000034793)

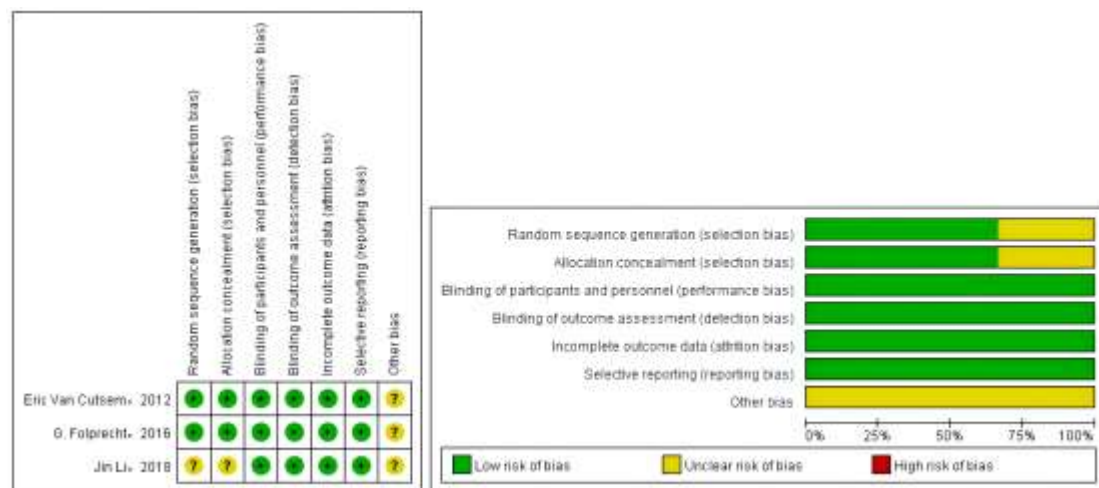

**Supplementary Figure 1** Assessment of risk of bias of included RCTS

Supplement: Supplementary file 5 [file medi-102-e34793-s005.pdf]
