# Supplementary material for: Risk of antiangiogenic adverse events in metastatic colorectal cancer patients receiving aflibercept in combination with chemotherapy: A meta-analysis
Source: Medicine (Baltimore). 2023 Sep 1;102(35):e34793. doi: 10.1097/MD.0000000000034793 (PMC10476758; doi:10.1097/MD.0000000000034793)
Supplement: Supplementary file 6 [file medi-102-e34793-s006.pdf]

**Supplementary Table 5** Assessment of risk of bias of included single-arm clinical studies

| Items | John-2019 | Alexandra-2020 | George-2018 | Benoist-2019 | Alexios-2019 | Tadamichi-2018 | Rachel-2019 |
|---|---|---|---|---|---|---|---|
| A clearly stated aim | 2 | 2 | 2 | 2 | 2 | 2 | 2 |
| Inclusion of consecutive patients | 2 | 2 | 2 | 2 | 2 | 2 | 2 |
| Prospective collection of data | 2 | 2 | 2 | 2 | 2 | 2 | 2 |
| Endpoints appropriate to the aim of the study | 2 | 2 | 2 | 2 | 2 | 2 | 2 |
| Unbiased assessment of the study endpoint | 2 | 2 | 2 | 2 | 2 | 2 | 2 |
| Follow- up period appropriate to the aim of the study | 1 | 2 | 2 | 2 | 2 | 2 | 2 |
| Loss to follow up less than 5% | 2 | 2 | 2 | 2 | 1 | 2 | 2 |
| Prospective calculation of the study size | 0 | 2 | 2 | 2 | 2 | 2 | 0 |

| | | | | | | | |
|---|---|---|---|---|---|---|---|
| Total scores | 13 | 16 | 16 | 16 | 15 | 16 | 14 |
